# Addressing the environmental and health impacts of microplastics requires open collaboration between diverse sectors

**DOI:** 10.1371/journal.pbio.3000932

**Published:** 2021-03-30

**Authors:** Scott Coffin, Holly Wyer, J. C. Leapman

**Affiliations:** 1 Division of Drinking Water, State Water Resources Control Board, California Environmental Protection Agency, Sacramento, California, United States of America; 2 Ocean Protection Council, California Environmental Protection Agency, Sacramento, California, United States of America; 3 School of Education, University of California, Davis, Davis, California, United States of America; Bennington College, UNITED STATES

## Abstract

Public concern over the environmental and public health impacts of the emerging contaminant class “microplastics” has recently prompted government agencies to consider mitigation efforts. Microplastics do not easily fit within traditional risk-based regulatory frameworks because their persistence and extreme diversity (of size, shape, and chemical properties associated with sorbed chemicals) result in high levels of uncertainty in hazard and exposure estimates. Due to these serious complexities, addressing microplastics’ impacts requires open collaboration between scientists, regulators, and policymakers. Here we describe ongoing international mitigation efforts, with California as a case study, and draw lessons from a similarly diverse and environmentally persistent class of emerging contaminants (per- and polyfluoroalkyl substances) that is already disrupting traditional regulatory paradigms, discuss strategies to address challenges associated with developing health-protective regulations and policies related to microplastics, and suggest ways to maximize impacts of research.

## Abstract 2

Mounting concerns regarding the deleterious effects of plastic pollution have prompted recent government mitigation efforts, but microplastics challenge traditional risk-based regulatory frameworks due to their particle properties, diverse composition and persistence. Using California as a case study, the Essay suggests strategies to address challenges with regulations, policies and research, drawing parallels with a similar class of emerging contaminants (per- and polyfluoroalkyl substances).

## Introduction

Recent polls suggest the public is aware of and concerned about the effects of plastic pollution on the environment and public health [1–3]. Microplastics (typically defined as plastic particles smaller than 5 mm [4]) are found virtually everywhere, including in aquatic and terrestrial ecosystems [5,6], air [7], drinking water [8], food [9], and even remote alpine and polar settings [10,11]. Adverse impacts of plastic pollution, particularly microplastics, are becoming better understood in aquatic ecosystems [12,13], with exceedances of risk thresholds documented in several ecosystems [14]. However, uncertainties regarding impacts remain, largely due to uncharacterized hazards and sampling bias towards larger-sized particles (which are believed to be less toxic) [12,15]. Greater uncertainties remain in assessing impacts to humans, which have received far less research attention than ecological receptors [16].

Generally, the public relies on the government to address environmental issues and often promotes policy and regulatory actions through citizen’s groups and nongovernmental organizations [17]. Accordingly, regulators and policymakers around the world have taken various actions to mitigate environmental and public health impacts [18]. Microplastics present unique challenges to risk assessors and decision-makers due to their extreme diversity of composition [19], insolubility, adsorbed and intentionally added contaminants [20], and complex, heterogeneous occurrence in the environment [21].

Despite these challenges, government agencies around the world are implementing various actions to mitigate known and unknown impacts of microplastics on public health and the environment. These actions range from upstream measures, such as Taiwan’s ban on single-use plastics [22], to downstream measures such as California’s discharge requirements of macro-sized debris into waterways [23]. While local and national efforts to reduce impacts of microplastics are valuable, international strategies and reduction targets such as the 1978 Protocol to the International Convention for the Preservation of Pollution from Ships (MARPOL) [24] are needed to significantly mitigate impacts [25]. In addition to the need for international cooperation in addressing impacts of microplastics, close intersector collaboration between scientists, regulators, and policymakers is paramount to advance policy and mitigation options available to local and national governments to reduce microplastic emissions. Such collaborative efforts may be exemplified in the State of California, which recently has enacted 2 groundbreaking pieces of legislation to address impacts of microplastics in drinking water and the marine environment to respond to increasing public concern [26,27].

This paper highlights several aspects of microplastics, which present unprecedented challenges for mitigating impacts, thus requiring close collaboration between stakeholders; uses California as a case study to offer insights on addressing some of these issues; and identifies actions that regulators, policymakers, and researchers can take to advance the field and develop effective pollution intervention strategies. Throughout this paper, we will refer to another regulatory paradigm disruptor—per- and polyfluoroalkyl substances (PFAS)—for insight and lessons learned when addressing microplastics.

### Microplastics challenge traditional risk-based regulatory paradigms

Innovations in risk assessment frameworks and regulatory approaches may be required to protect environmental and public health from complex contaminant classes and mixtures with vast uncertainties in their environmental fate and transport, exposure, and hazards. The traditional framework for assessing risk is by comparing exposure amounts with known hazard thresholds [28]. Many regulatory frameworks are based on this traditional risk assessment framework and set regulatory thresholds (e.g., maximum contaminant levels in drinking water, effluent limits in wastewater discharge) based on estimated exposures which would theoretically exceed certain risk thresholds [29,30]. While this traditional risk assessment-based regulatory framework works well for single-chemical contaminants or relatively simple mixtures of contaminants with known chemical structures, compositions, and biological activities (e.g., dioxins and dioxin-like polychlorinated biphenyls (PCBs) [10]), it may be inadequate to address risks from more complex contaminants.

The term “microplastics” encompasses a vast universe of particles that present unique challenges in estimating risks due to their extreme diversity (e.g., size, shape, solubility, polymer composition, sorbed chemicals and biota, etc.) [19]. Even defining the contaminant class has been a matter of lengthy debate [4,31]. In order to estimate risks and regulate microplastics using traditional frameworks, recent innovative efforts have tried to reduce complexities associated with the high number of variables used to classify microplastics (i.e., size, shape, polymer types) [32]. However, such simplification efforts are unlikely to satisfactorily capture the full variability of shapes of microplastics, leading to underestimates of risk [32].

A recent study on wild-caught, commercially important fish found that microplastics ingested by the fish likely transferred bisphenol A (BPA) and related analogues into their tissue at high enough quantities to exceed risk thresholds in adults and children at mean ingestion rates of the fish [33]. Notably, the study would not have estimated an exceedance of risk threshold if the authors had used the United States Environmental Protection Agency’s (US EPA) risk value for BPA [34], which is 12.5 times higher than the European Food Safety Authority’s value [35]. This study highlights both the importance of characterizing plastic-associated chemicals in microplastics (a key hazard trait) [36–39] and assessing hazards of endocrine-disrupting chemicals (e.g., BPA, di-2-ethylhexyl-phthalate) commonly added to plastics [37,40]. Assessing hazards for some endocrine-disrupting chemicals may be complicated due their exhibiting nonmonotonic dose–response effects (i.e., effects observed at low concentrations are not predicted by and/or observed at higher concentrations) [41–43]. When such nonmonotonic effects are considered, such compounds may be considered substantially more toxic [44].

Another critical challenge in assessing risks of plastic-associated chemicals is that most plastic additives (approximately 80%) have their identities hidden from researchers, regulators, and the public, protected as “confidential business information” (CBI), or lack adequate documentation in public databases (see more on unknown chemicals in Box 1) [45,46]. Additionally, complex mixtures of chemicals on microplastics may exhibit mixture toxicity effects (i.e., additive, synergistic, antagonistic) [47], making their identification complicated [20].

**Box 1. Unknown chemicals present never-ending challenges for risk assessors**

There is an increasing worldwide trend of approving unknown chemicals and mixtures for use in commerce, thus providing scientists and regulators with a Sisyphean task in estimating risks for over 70,000 such chemicals/mixtures (>37,000 of which are polymers) [46]. This increasing trend is in spite of regulations that apparently intend to prevent the introduction of “regrettable substitutions” into the environment, such as the 2016-revised Toxic Substances Control Act (TSCA) in the United States (US) [48] and the European Union’s more aggressive Registration, Evaluation, Authorisation, and Restriction of Chemicals [49]. Yet chemicals/mixtures protected as CBI lack information regarding chemical structure, composition and biological activities, and access to analytical standards [47], requiring innovative methods to determine hazardous chemical features within an unknown plastic chemical mixture, such as bioassay-guided chemical fractionation coupled with nontargeted analytical chemistry [20,45]. Such techniques are costly, however, and it remains unlikely that risks could ever be characterized with a high degree of certainty until full chemical compositions are known. Voluntary cooperation between industry and researchers in revealing the identity of some of these CBI chemicals provides a possible avenue for reducing such uncertainties [50].

In addition to complicating the assessment of risk for chemicals and mixtures already present in the environment, protections provided by CBI may lead to the continued introduction of potentially hazardous chemicals into the environment [51]. For example, in 2018, the identities, quantities produced, location of production facilities, and other data for 396 new PFAS was withheld by manufacturers on the basis that such information is CBI [52]. Such confidential compounds may eventually be characterized years later using nontargeted analytical chemistry, as demonstrated by the recent discovery of a new class of chlorinated PFAS (apparently used as a substitute for other banned PFAS [53])—chloroperfluoropolyether carboxylate compounds (ClPFPECAs) [54]. Most concerning, ClPFPECAs are considered to be safe for use in polymerized nonstick cookware by the European Food Safety Authority [55]—despite their similarities to other PFAS, and a complete lack of publicly available toxicity information [56]. ClPFPECAs are unregistered in both the US EPA’s and the European Chemical Agency’s inventories [56]. It’s possible that ClPFPECAs passed EPA’s review without much, or any toxicity testing, as under TSCA (pre-2016 amendment), EPA was required to produce evidence for potential risk in order to investigate a chemical further [48,49]—a catch-22 that allowed 90% of chemicals entering commerce between 1979 and 2016 to evade restrictions or testing orders [56].

The extremely diverse nature of microplastics is unparalleled; however, another emerging contaminant class may come relatively close and may provide insights for risk management. PFAS, like microplastics, are persistent, toxic, and largely unregistered in regulatory inventories [46,57,58]. The push to regulate PFAS in a timely manner has prompted some scientists and regulators to develop alternative methods to estimate their exposure and determine their hazards to estimate risk. A recent study estimated that there are over 4,700 PFAS chemicals distributed in the global market [59]—a multiplicity that makes developing analytical methods and determining toxicological effects for all constituents unachievable within reasonable timeframes. Novel, proxy-based approaches have been developed to estimate exposure (e.g., total fluorine [60,61]), and 21st century approaches are being applied to characterize hazards of PFAS (e.g., read-across [62]). Some of these approaches have proven, in some cases, to be health protective and simple, and are being considered for adoption by regulatory agencies [63–65]. While PFAS provide lessons for addressing extremely diverse and unique contaminant classes, microplastics are likely more complex and challenging (Box 2).

**Box 2. Microplastics are a more complex contaminant class than PFAS**

While many similarities exist between PFAS and microplastics (e.g., persistence, diversity, unknown composition, bioaccumulative potential, toxicity), there are principal differences between these contaminant classes which make understanding risks of microplastics arguably more challenging. The principal difference is that PFAS (with the exception of polymers and anions) are generally soluble [66], while microplastics are (generally) insoluble [67]—thus having distinct physicochemical properties that may drive toxicological behavior as well as fate and transport characteristics—all of which are foundational in assessing exposure and risks. For other “conventional contaminants” (e.g., petroleum hydrocarbons), fate and transport characteristics are well studied [68]. Due to the diversity of the PFAS class and their unique characteristics (hydrophobic, lipophilic, and surfactant properties), traditional fate and transport models have proven inadequate in modeling their behavior—particularly in groundwater [68,69]. Even less understood are the environmental fate and transport behavior of microplastic particles, in which key determining factors are unique to insoluble particles (relatively less studied than soluble contaminants) and, in some cases, unique to synthetic polymers, such as: formation and emissions of microplastic particles; particle–particle interactions (e.g., aggregation and agglomeration); biological uptake and bioaccumulation; and transport via air and oceanic circulation [70]. Significant challenges for testing the toxicity of dispersed particles in aqueous systems remain [71], and extrapolating effects of exposure at high concentrations to lower, environmentally relevant concentrations may not be appropriate [70]. Further challenges in assessing microplastics toxicity are the lack of standardized, environmentally realistic mixture samples, and the selection of natural particles as controls [72]. Finally, determining the drivers of microplastics toxicity (e.g., physical, chemical) is difficult [73], as exemplified by the association of PFAS with plastic [74].

### Plastics and PFAS are forever

In an effort to prevent irreversible damage from persistent chemicals with poorly known effects, some regulatory agencies in Europe and the US have departed from their traditional risk-based frameworks. They are doing so by taking a more precautionary approach, classifying certain chemicals as “nonthreshold contaminants” (i.e., “any release to the environment and environmental monitoring data regarded as a proxy for an unacceptable risk”) [75,76]. A critical driver behind considerations of such precautionary management approaches is a chemical’s ability to resist degradation in the environment (persistence) [77]—a trait which is shared by both microplastics and PFAS [55,75]. The combination of global environmental contamination, persistence, and uncertainties regarding effects on vital earth system processes satisfy the conditions for both PFAS and microplastics to be classified as “planetary boundary threats”—defined as factors that may irreversibly threaten the earth systems that allow humanity to thrive [78,79]. Indeed, PFAS are often referred to as “Forever Chemicals”—implying that their persistence should be worrying [80].

In 2019, Denmark banned all PFAS (known and unknown) in paper and cardboard food contact materials [81]. This broad, class-based restriction was aimed at preventing widespread, irreversible environmental contamination of persistent, bioaccumulative, toxic chemicals within the PFAS class [57]. In managing PFAS, the concept of “essential use” is integral to drafting sensible, risk-based restriction regulations [82]—an approach which has been considered by the European Chemicals Agency in restricting the use of intentionally added microplastics [75], and may also be useful in considering restrictions of single-use plastic products in a circular economy.

Like PFAS, microplastics are ubiquitous in the environment [83], and some particle types are known to be toxic and bioaccumulative [9,58], thus concerns over environmental persistence [84] are warranted [85,86]. With the continuous production and release of persistent chemicals, risk thresholds are likely to be exceeded over time, regardless of the chemical’s properties [77]. This high likelihood of eventually exceeding risk thresholds renders traditional risk assessments inadequate, as they typically do not consider long-term impacts to future generations, or system-level effects at regional (or even global) scales.

The San Francisco Bay Regional Monitoring Program, which ranks contaminants of emerging concern monitored in water, sediment, and biota into tiered, risk-based categories (based on occurrence and hazard ratios) [76], initially classified microplastics as a constituent class of “Possible Concern” based on uncertainties regarding toxicity, but later elevated microplastics to “Moderate Concern,” despite a noted lack of certainty regarding hazard thresholds [87]. The San Francisco Bay Regional Monitoring Program justified this departure from their established risk-based framework based on the EU’s decision to classify microplastics as a nonthreshold contaminant for risk assessment purposes [75]; uncertainties regarding toxicities [87]; an upward trend in both plastic production and environmental detection [88–90]; and persistence [75,87]. These decisions are in congruence with conclusions made by the Science Advice for Policy by European Academies, which state that while risk thresholds are exceeded at some locations (i.e., predicted or measured concentrations are greater than predicted no-effect levels), it is unlikely that exceedances of risk thresholds are geographically widespread [12]; however with expected increases in exposure to microplastics [91], widespread ecological risk may arise within the next century [12]. In other words, while traditional regulatory frameworks typically focus on short-term risks from chemicals with known hazards, highly complex, persistent contaminants with unknown hazards are being recognized as potential irreversible global scale threats and are being precautionarily evaluated by regulators and scientists.

### A case study for intersector collaboration: California legislation as a regulation, policy, and science driver

**Fig 1:**
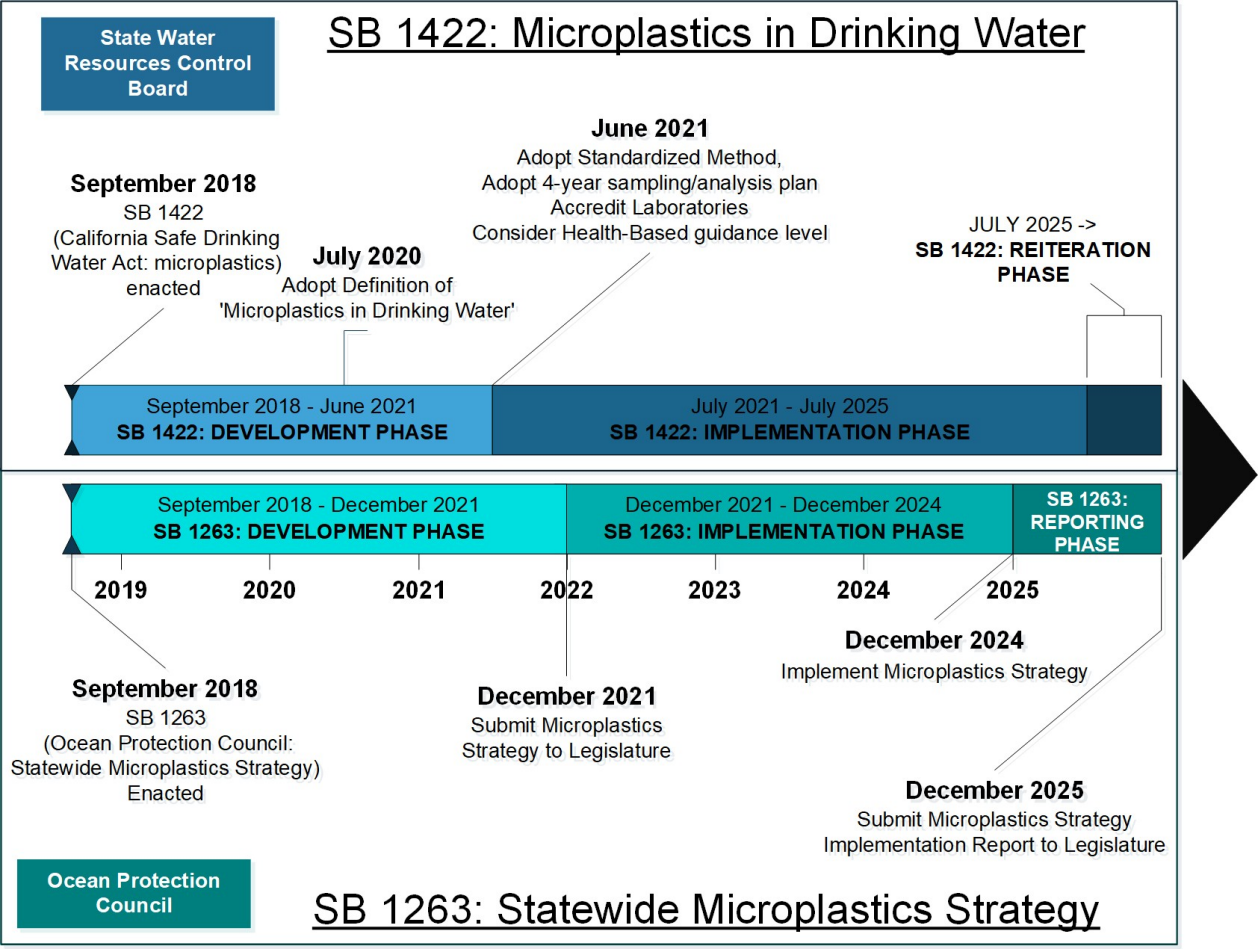
Timelines for implementation of California Senate Bills 1422 and 1263. Requirements and timeline for implementation of recently passed California legislation aimed at advancing understandings of microplastics in drinking water (Senate Bill 1422) and in marine ecosystems (Senate Bill 1263). The California Ocean Protection Council, in collaboration with the State Water Resources Control Board, must implement requirements of Senate Bill 1263. The State Water Resources Control Board will implement requirements of Senate Bill 1422.

California Senate Bills (SB) 1422 and SB 1263 outline initial steps to address microplastics in drinking water and the ambient marine environment, respectively [26,27]. In response to initial findings of widespread contamination of drinking water with microplastics [86] and considerable uncertainties regarding their health risks to humans at the time [92], the California Legislature passed SB 1422 in 2018, which requires the State Water Resources Control Board (State Water Board) to adopt a definition for “microplastics in drinking water” by July 1, 2020 (see Box 3 for more), and to adopt a standard methodology for detecting microplastics in drinking water by July 1, 2021 (Fig 1). Additionally, the bill requires 4 years of testing and reporting of microplastics in drinking water, public disclosure of the results, and possible issuance of a health-based guidance level to interpret results [26]. SB 1263 requires the California Ocean Protection Council to adopt a statewide microplastics strategy (Strategy) [27]. The Strategy shall include the development of standardized methods for sampling, detecting, and characterizing microplastics, development of a risk assessment framework for microplastics, and the use of that risk assessment framework to identify data gaps, and effective policy changes to reduce risks due to microplastic pollution in the ambient marine environment (Fig 1) [27].

To meet unprecedented challenges in addressing perhaps the most complex, diverse, and publicly visibly contaminant suite (plastics pollution; including microplastics), California is partnering with an international network of researchers, local, state, and federal agencies, nongovernmental organizations, water purveyors, and engaged citizens. For example, the California State Water Board has a long history of working with citizen scientists regarding characterizing trash and microplastics in water, reporting some of the earliest findings of persistent organic pollutants on preproduction plastics pellets along California’s beaches in 2005 [93]. Starting in 2018, the California State Water Board began hosting annual, multiday, immersive “Trash Data Dives” where researchers (data scientists and trash/microplastic experts) work alongside municipalities, policy writers, regulators, nongovernmental organization leaders, community leaders, and others to develop a, “trash management picture informed by open and accessible data, to identify and understand trends, data gaps, and priorities” [94]. Similarly, the California Ocean Protection Council (which has made policy recommendations to reduce plastic pollution since 2007 [95]) partnered with the National Oceanic and Atmospheric Administration in 2018 to develop the California Ocean Litter Prevention Strategy, which outlines actions that California and interested stakeholders can take to address ocean litter through 2024 [96]. The Ocean Litter Prevention Strategy laid out critical needs in microplastics research, such as standardized methods, which were later included in SB 1263 [96].

In implementing SB 1422 and SB 1263, the California Ocean Protection Council and California State Water Board are collaborating with a wide range of stakeholders to accomplish the ambitious objectives required by the bills. The public research and development agency, the Southern California Coastal Water Research Project (SCCWRP) plays a pivotal role in the State’s microplastics-related projects, coordinating more than 35 laboratories based in 7 different countries to standardize microplastics monitoring methods in aquatic environments, and serving as facilitator for the development of a consensus statement on the human health effects of microplastics in drinking water [97]. Additionally, the Ocean Protection Council is collaborating with an independent science-based nonprofit, The Ocean Science Trust, to convene an internationally recognized expert panel to develop a microplastics risk assessment framework as part of their Strategy [98]. Intersector working groups, such as the Pacific Northwest Consortium on Plastics and San Francisco Estuary Institute Microplastics Working Group, play key roles in coordinating local and regional research efforts that directly inform decision makers, and serve as exemplary models for constructive interactions between policymakers, scientists, regulators, and industry representatives.

**Box 3. Lessons learned from PFAS in developing a regulatory definition for microplastics**

After the discovery of some fluorinated chemicals in food contact materials (e.g., perfluoropolyether dicarboxylic acid) that were not formally recognized as PFAS under their definition at the time (-C_n_F2_n+1_-) [59,99], the Organisation for Economic Co-operation and Development (OECD) expanded their definition (-C_n_F2_n_-) [59]. Meanwhile, other organizations (e.g., Interstate Technology Regulatory Council) more narrowly define perfluoroalkyl substances as having two or more fully fluorinated carbons (-C_n_F2_n+1_-), and polyfluoroalkyl substances as having a nonfluorine atom (typically hydrogen or oxygen) attached to at least one, but not all, carbon atoms, with at least two or more fully fluorinated carbons (-C_n_F2_n+1_-), with a further explicit exclusion of aromatic carbon ring substances [66]. In the case of extremely environmentally persistent chemicals like PFAS, the exclusion of certain chemicals from the contaminant class has resulted in a systematic lack of focus on their existence—resulting in sparse monitoring data (e.g., aromatic carbon ring PFAS) [100].

This debacle demonstrates the importance of starting with a broad definition as a common departure point for further definitions for microplastics and other emerging contaminant classes with significant uncertainties. Failure to start with a broad definition and consider all constituents within the class has resulted in the likely human exposure to short- (4 to 7 carbons) and ultrashort-chain (2 to 3 carbons) PFAS through food packaging in the US (e.g., 1,1,1,2-tetrafluoroethane, which is “generally recognized as safe” by the nation’s Food and Drug Administration) [101]. With regulatory agencies focused on long-chain PFAS (8+ carbons), industry has increased production of short and ultrashort alternatives [102], even though they were included in the once commonly accepted definition of PFAS (-C_n_F_2n+1_-). Learning from mistakes made with PFAS, if regulatory definitions of microplastics are too narrow, risks may be underestimated due to their incomplete characterization and lack of consideration for the vast possibilities within the contaminant class.

A challenge in implementing California’s legislative requirements to address microplastics in drinking water (SB 1422) was the apparent lack of a consensus definition for “microplastics.” Despite calls for a unified, internationally agreed-upon definition for “microplastics” [103], it seems that no such definition had emerged due (in part) to the lack of both standardized methods and regulations. Due to the regulatory impacts (i.e., monitoring and reporting and communicating health effects to consumers) associated with adopting a definition of microplastics in the context of drinking water, California’s State Water Board recognized that the definition they adopted in June 2020 would likely be used for nondrinking water purposes and by other government agencies and scientific bodies [104]. In drafting an initial regulatory definition for microplastics (which have extreme uncertainties in regards to exposure and hazards for humans [105]), a principal consideration was to use terms that broadly encompass particle sizes (1 nm to 5 mm), types (e.g., theoretically soluble plastics), and polymers (e.g., including biodegradable polymers, for which limited toxicity information is available [106]) to avoid inappropriately restricting risk assessments based on regulatory definitions [70], as well as research, monitoring, and collection of data—at least until the adoption of a more narrow definition can be justified [31]. Drawing lessons learned from PFAS, subcategories of microplastics may be grouped for strategic purposes for monitoring and regulations [107], however should be distinguished from a broader class-based definition, with exclusions and limitations acknowledged wherever possible [108].

### Making microplastics research “actionable”: Standardized methods and beyond

Scientific organizations have long called for the standardization of microplastic analysis methods [109,110]; the legislative requirements for California to adopt standard methodologies to monitor microplastics provides an impetus and requisite funding to develop such methods [26,27]. Standardization of microplastic monitoring methods will allow for direct comparisons between studies, may reduce uncertainties in assessments of risk, and reliably inform management strategies. It is important to keep in mind that unintended consequences may result if practical considerations of enacting regulations inhibit broader research investigations. For example, standardized methodologies may miss certain components (e.g. < approximately 10 μm particles, black particles) due to technical and economic barriers—a phenomenon that has caused a significant mismatch in the size ranges of particles used in toxicological assessments and monitored in the environment [15]. Therefore, as regulatory agencies adopt standardized methods for analyzing microplastics, the academic community should continue to improve detection methodologies [111], and regulatory agencies should consider regularly updating their standardized methods.

In addition to developing standardized methods for monitoring microplastics in the environment, food, and water, further research is necessary to develop evidence-based policies and regulations. The policy and regulatory communities need actionable research that focuses on (a) addressing gaps in the understanding of the ecological and human health hazards and exposure of microplastics; (b) identifying and prioritizing sources (e.g., packaging, tire wear, textiles) and pathways (e.g., washing machines, stormwater, wastewater, biosolid agriculture application) that may be candidates for regulatory intervention; and (c) developing cost-effective technologies to reduce economic impacts of policy and regulatory interventions (e.g., analysis methods, water treatment, reusable or truly biodegradable materials). Moreover, quantitative toxicological risk assessments may be necessary under certain regulatory paradigms to effectively regulate microplastics as a water quality contaminant [112]. A useful strategy to accurately assess and convey risks associated with plastic without downplaying the potential of uncertain risks is to focus on known particle- and species-specific effect mechanisms (e.g., adverse outcome pathways) [112]. These adverse outcome pathways allow for the separation of hazards of plastic-associated chemicals with the physical particles themselves [112], allowing for a more simplistic understanding and communication of risks and development of risk-based regulations and policies. Most regulatory paradigms will prioritize high-risk microplastic morphologies—thus research should focus on reducing toxicological dimensions of complex mixtures to simplify sampling and monitoring plans [32]. Finally, research findings should be written so that they can be easily summarized and distilled into fact sheets and talking points, which are useful for both general media inquiries and policy briefings.

## Conclusion

Microplastics as a contaminant class are unmatched in their magnitude of complexity, diversity, and persistence (with PFAS likely being the closest in all 3 categories), presenting significant challenges for scientists in developing analytical methods, fate and transport models, characterization of exposure pathways, and assessment of toxicological hazards. Considering unprecedented uncertainties associated with risks to humans and ecosystems, governmental organizations are reconsidering the appropriateness of applying traditional frameworks in mitigating risks of microplastics (and PFAS), opting in some cases for more precautionary approaches that give additional weight to uncertainties and environmental persistence. To address such challenging and complex emerging contaminant classes, governments should coordinate closely with researchers, citizens, industry representatives, and commercial monitoring laboratories, and should actively promote transparency, data accessibility, and civic engagement. California’s pioneering efforts in addressing microplastics in drinking water and aquatic ecosystems serves as a model for developing open collaborations between diverse sectors.

## Abbreviations

BPA: bisphenol A
CBI: confidential business information
ClPFPECA: chloroperfluoropolyether carboxylate compound
OECD: Organisation for Economic Co-operation and Development
PCB: polychlorinated biphenyl
PFAS: per- and polyfluoroalkyl substances
SB: Senate Bill
SCCWRP: Southern California Coastal Water Research Project
TSCA: Toxic Substances Control Act
US EPA: United States Environmental Protection Agency

## References

[1] Dilkes-Hoffman LS, Pratt S, Laycock B, Ashworth P, Lant PA. Public attitudes towards plastics. Resour Conserv Recycl. 2019;147: 227–235. 10.1016/j.resconrec.2019.05.005

[2] European Commission. Special Eurobarometer 468: Attitudes of European citizens towards the environment. 2017. Available from: http://ec.europa.eu/commfrontoffice/publicopinion/index.cfm/ResultDoc/download/DocumentKy/81259.

[3] Lotze HK, Guest H, O’Leary J, Tuda A, Wallace D. Public perceptions of marine threats and protection from around the world. Ocean & Coastal Management. 2018;152: 14–22. 10.1016/j.ocecoaman.2017.11.004

[4] Hartmann NB, Hüffer T, Thompson RC, Hassellöv M, Verschoor A, Daugaard AE, et al. Are We Speaking the Same Language? Recommendations for a Definition and Categorization Framework for Plastic Debris. Environ Sci Technol. 2019;53: 1039–1047. 10.1021/acs.est.8b05297 30608663

[5] Suaria G, Achtypi A, Perold V, Lee JR, Pierucci A, Bornman TG, et al. Microfibers in oceanic surface waters: A global characterization. Sci Adv. 2020;6: eaay8493. 10.1126/sciadv.aay8493 32548254 PMC7274779

[6] Hurley R, Horton A, Lusher A, Nizzetto L. Chapter 7—Plastic waste in the terrestrial environment. In: Letcher TM, editor. Plastic Waste and Recycling. Academic Press; 2020. pp. 163–193. 10.1016/B978-0-12-817880-5.00007–4

[7] Gaston E, Woo M, Steele C, Sukumaran S, Anderson S. Microplastics Differ Between Indoor and Outdoor Air Masses: Insights from Multiple Microscopy Methodologies. Appl Spectrosc. 2020; 000370282092065. 10.1177/0003702820920652 32233850

[8] Shen M, Song B, Zhu Y, Zeng G, Zhang Y, Yang Y, et al. Removal of microplastics via drinking water treatment: Current knowledge and future directions. Chemosphere. 2020;251: 126612. 10.1016/j.chemosphere.2020.126612 32443234

[9] van Raamsdonk LWD, van der Zande M, Koelmans AA, Hoogenboom RLAP, Peters RJB, Groot MJ, et al. Current Insights into Monitoring, Bioaccumulation, and Potential Health Effects of Microplastics Present in the Food Chain. Foods. 2020;9: 72. 10.3390/foods9010072 31936455 PMC7022559

[10] Allen S, Allen D, Phoenix VR, Le Roux G, Durántez Jiménez P, Simonneau A, et al. Atmospheric transport and deposition of microplastics in a remote mountain catchment. Nat Geosci. 2019;12: 339–344. 10.1038/s41561-019-0335-5

[11] Bergami E, Rota E, Caruso T, Birarda G, Vaccari L, Corsi I. Plastics everywhere: first evidence of polystyrene fragments inside the common Antarctic collembolan *Cryptopygus antarcticus*. Biol Lett. 2020;16: 20200093. 10.1098/rsbl.2020.0093 32574531 PMC7336848

[12] Science Advice for Policy by European Academies. A Scientific Perspective on Microplastics in Nature and Society. Berlin; 2019 Jan. Report No.: 978-3-9820301-0–4. 10.26356/microplastics

[13] Bucci K, Tulio M, Rochman C. What is known and unknown about the effects of plastic pollution: A meta-analysis and systematic review. Ecol Appl. 2019; eap.2044. 10.1002/eap.2044 31758826

[14] Burns EE, Boxall ABA. Microplastics in the aquatic environment: Evidence for or against adverse impacts and major knowledge gaps: Microplastics in the environment. Environ Toxicol Chem. 2018;37: 2776–2796. 10.1002/etc.4268 30328173

[15] Adam V, Yang T, Nowack B. Toward an ecotoxicological risk assessment of microplastics: Comparison of available hazard and exposure data in freshwaters. Environ Toxicol Chem. 2019;38: 436–447. 10.1002/etc.4323 30488983 PMC6849787

[16] Zhang Y, Pu S, Lv X, Gao Y, Ge L. Global trends and prospects in microplastics research: A bibliometric analysis. J Hazard Mater. 2020;400: 123110. 10.1016/j.jhazmat.2020.123110 32574874

[17] Beierle TC. Democracy in practice: Public participation in environmental decisions. Routledge; 2010.

[18] United Nations Environment. Combating marine plastic litter and microplastics: An Assessment of the Effectiveness of Relevant International, Regional and Subregional Governance Strategies and Approaches. AHEG/2018/1/INF/3 (11 April 2018) 12 (UNEP ‘Assessment Report’); 2017. Available from: https://papersmart.unon.org/resolution/uploads/unep_aheg_2018_1_inf_3_summary_policy_makers.pdf.

[19] Rochman CM, Brookson C, Bikker J, Djuric N, Earn A, Bucci K, et al. Rethinking microplastics as a diverse contaminant suite. Environ Toxicol Chem. 2019;38: 703–711. 10.1002/etc.4371 30909321

[20] Chen Q, Santos MM dos, Tanabe P, Harraka GT, Magnuson JT, McGruer V, et al. Bioassay guided analysis coupled with non-target chemical screening in polyethylene plastic shopping bag fragments after exposure to simulated gastric juice of Fish. J Hazard Mater. 2021;401: 123421. 10.1016/j.jhazmat.2020.123421 32763709

[21] Skåre JU, Alexander J, Haave M, Jakubowicz I, Knutsen HK, Lusher A, et al. Microplastics; occurrence, levels and implications for environment and human health related to food. Scientific opinion of the Scientific Steering Committee of the Norwegian Scientific Committee for Food and Environment. VKM Report. 2019.

[22] News Taiwan. Taiwan wages war on single-use plastics. 2020.

[23] State Water Resources Control Board. Amendment to the Water Quality Control Plan for Ocean waters of California to Control Trash and Part 1 Trash Provisions of the Water Quality Control Plan for Inland Surface Waters, Enclosed Bays, and Estuaries of California. 2016.

[24] Canyon T. International Convention for the Prevention of Pollution from Ships, 1973, as modified by the Protocol of 1978 relating thereto (MARPOL 73/78). 1978.

[25] Simon N, Schulte ML. Stopping global plastic pollution: The case for an international convention. Ecology Publication Series. 2017;43.

[26] California Code of Regulations. California Safe Drinking Water Act. Health and Safety Code 116350. Health and Safety Code 2018.

[27] California Code of Regulations. Microplastics Materials. Sect. 1 2018. Available from: https://leginfo.legislature.ca.gov/faces/billTextClient.xhtml?bill_id=201720180SB1263.

[28] United States Environmental Protection Agency. Guidelines for the health risk assessment of chemical mixtures. Fed Reg. 1986;51: 34014–34025.

[29] US Environmental Protection Agency. National primary drinking water regulations; final rule. 40 CFR Parts 141, 142, and 143. Federal Register. 1991; 3526–3597.

[30] United States. “Effluent Limitations.” Clean Water Act (CWA), section 301, 33 U.S.C. § 1311. 1972.

[31] Coffin S. Staff Report for the Proposed Definition of Microplastics in Drinking Water (June 3, 2020). Sacramento, CA: State Water Resources Control Board; 2020 6. Available from: https://www.waterboards.ca.gov/drinking_water/certlic/drinkingwater/docs/stffrprt_jun3.pdf.

[32] Kooi M, Koelmans AA. Simplifying Microplastic via Continuous Probability Distributions for Size, Shape, and Density. Environ Sci Technol Lett. 2019;6: 551–557. 10.1021/acs.estlett.9b00379

[33] Barboza LGA, Cunha SC, Monteiro C, Fernandes JO, Guilhermino L. Bisphenol A and its analogs in muscle and liver of fish from the North East Atlantic Ocean in relation to microplastic contamination. Exposure and risk to human consumers. J Hazard Mater. 2020; 122419. 10.1016/j.jhazmat.2020.122419 32155522

[34] U.S. Environmental Protection Agency. Bisphenol A; CASRN 80-05-7. Integrated Risk Information System. Chemical Assessment Summary. 1988. Available from: https://cfpub.epa.gov/ncea/iris/iris_documents/documents/subst/0356_summary.pdf.

[35] EFSA Panel on Food Contact Materials E Flavourings and Processing Aids (CEF). Scientific opinion on the risks to public health related to the presence of bisphenol A (BPA) in foodstuffs. EFSA J. 2015;13: 3978.

[36] Koelmans AA, Besseling E, Foekema EM. Leaching of plastic additives to marine organisms. Environ Pollut. 2014;187: 49–54. 10.1016/j.envpol.2013.12.013 24440692

[37] Coffin S, Huang G-Y, Lee I, Schlenk D. Fish and Seabird Gut Conditions Enhance Desorption of Estrogenic Chemicals from Commonly-Ingested Plastic Items. Environ Sci Technol. 2019;53: 4588–4599. 10.1021/acs.est.8b07140 30905144

[38] Almeida S, Raposo A, Almeida-González M, Carrascosa C. Bisphenol A: Food Exposure and Impact on Human Health: Bisphenol A and human health effect…. Compr Rev Food Sci Food Saf. 2018;17: 1503–1517. 10.1111/1541-4337.12388 33350146

[39] Coffin S, Dudley S, Taylor A, Wolf D, Wang J, Lee I, et al. Comparisons of analytical chemistry and biological activities of extracts from North Pacific gyre plastics with UV-treated and untreated plastics using in vitro and in vivo models. Environ Int. 2018;121: 942–954. 10.1016/j.envint.2018.10.012 30352377

[40] Yang CZ, Yaniger SI, Jordan VC, Klein DJ, Bittner GD. Most plastic products release estrogenic chemicals: a potential health problem that can be solved. Environ Health Perspect. 2011;119: 989–996. 10.1289/ehp.1003220 21367689 PMC3222987

[41] Do RP, Stahlhut RW, Ponzi D, vom Saal FS, Taylor JA. Non-monotonic dose effects of in utero exposure to di (2-ethylhexyl) phthalate (DEHP) on testicular and serum testosterone and anogenital distance in male mouse fetuses. Reprod Toxicol. 2012;34: 614–621. 10.1016/j.reprotox.2012.09.006 23041310 PMC3543148

[42] National Toxicology Program. NTP research report on the CLARITY-BPA core study: a perinatal and chronic extended-dose-range study of bisphenol A in rats. 2018. 31305969

[43] Hill CE, Myers JP, Vandenberg LN. Nonmonotonic Dose–Response Curves Occur in Dose Ranges That Are Relevant to Regulatory Decision-Making. Dose-Response. 2018;16: 155932581879828. 10.1177/1559325818798282 30228814 PMC6137554

[44] Vandenberg LN, Colborn T, Hayes TB, Heindel JJ, Jacobs DR, Lee D-H, et al. Hormones and Endocrine-Disrupting Chemicals: Low-Dose Effects and Nonmonotonic Dose Responses. Endocr Rev. 2012;33: 378–455. 10.1210/er.2011-1050 22419778 PMC3365860

[45] Zimmermann L, Dierkes G, Ternes TA, Völker C, Wagner M. Benchmarking the in Vitro Toxicity and Chemical Composition of Plastic Consumer Products. Environ Sci Technol. 2019;53: 11467–11477. 10.1021/acs.est.9b02293 31380625

[46] Wang Z, Walker GW, Muir DCG, Nagatani-Yoshida K. Toward a Global Understanding of Chemical Pollution: A First Comprehensive Analysis of National and Regional Chemical Inventories. Environ Sci Technol. 2020;54: 2575–2584. 10.1021/acs.est.9b06379 31968937

[47] Muncke J, Backhaus T, Geueke B, Maffini MV, Martin OV, Myers JP, et al. Scientific challenges in the risk assessment of food contact materials. Environ Health Perspect. 2017;125: 095001. 10.1289/EHP644 28893723 PMC5915200

[48] Watnick VJ. The Lautenberg Chemical Safety Act of 2016: Cancer, Industry Pressure, and a Proactive Approach. Harv Envtl L Rev. 2019;43: 373.

[49] Applegate JS. Synthesizing TSCA and REACH: practical principles for chemical regulation reform. Ecol Law Q. 2008;35: 721.

[50] Frond HL, Sebille E, Parnis JM, Diamond ML, Mallos N, Kingsbury T, et al. Estimating the Mass of Chemicals Associated with Ocean Plastic Pollution to Inform Mitigation Efforts. Integr Environ Assess Manag. 2019;15: 596–606. 10.1002/ieam.4147 30900806

[51] Sheriff I, Debela SA, Kabia OA, Ntoutoume CE, Turay MJ. The phase out of and restrictions on per-and polyfluoroalkyl substances: Time for a rethink. Chemosphere. 2020;251: 126313. 10.1016/j.chemosphere.2020.126313 32143075

[52] Lerner S. EPA continues to approve toxic PFAS chemicals despite widespread contamination. The Intercept. 201825. Available from: https://theintercept.com/2018/10/25/epa-pfoa-pfas-pfos-chemicals/.

[53] US EPA. EPA docket on PFOA voluntary stewardship program, docket number EPA-HQ-OPPT-2006-0621; 2006. Available from: https://www.regulations.gov/document?D=EPA-HQOPPT-2006-0621-0005.

[54] Washington JW, Rosal CG, McCord JP, Strynar MJ, Lindstrom AB, Bergman EL, et al. Nontargeted mass-spectral detection of chloroperfluoropolyether carboxylates in New Jersey soils. Science. 2020;368: 1103–1107. 10.1126/science.aba7127 32499438 PMC7814412

[55] EFSA Panel on food contact materials enzymes flavourings and processing aids (CEF). Scientific Opinion on the safety evaluation of the substance perfluoro acetic acid, α-substituted with the copolymer of perfluoro-1, 2-propylene glycol and perfluoro-1, 1-ethylene glycol, terminated with chlorohexafluoropropyloxy groups, CAS No. 329238–24–6 for use in food contact materials. EFSA J. 2010;8: 1519.

[56] Gold SC, Wagner WE. Filling gaps in science exposes gaps in chemical regulation. Science. 2020;368: 1066–1068. 10.1126/science.abc1250 32499431

[57] Sunderland EM, Hu XC, Dassuncao C, Tokranov AK, Wagner CC, Allen JG. A review of the pathways of human exposure to poly- and perfluoroalkyl substances (PFASs) and present understanding of health effects. J Expo Sci Environ Epidemiol. 2019;29: 131–147. 10.1038/s41370-018-0094-1 30470793 PMC6380916

[58] Goswami P, Vinithkumar NV, Dharani G. First evidence of microplastics bioaccumulation by marine organisms in the Port Blair Bay, Andaman Islands. Mar Pollut Bull. 2020;155: 111163. 10.1016/j.marpolbul.2020.111163 32469778

[59] Organisation for Economic Co-operation and Development. Towards a New Comprehensive Global Database of Per-and Polyfluoroalkyl substances (PFASs): Summary Report on Updating the OECD 2007 List of Per- and Polyfluoroalkyl substances (PFASs). Series on Risk Management No. 39. 2018. Available from: http://www.oecd.org/officialdocuments/publicdisplaydocumentpdf/?cote=ENV-JM-MONO(2018)7&doclanguage=en.

[60] Ritter EE, Dickinson ME, Harron JP, Lunderberg DM, DeYoung PA, Robel AE, et al. PIGE as a screening tool for Per- and polyfluorinated substances in papers and textiles. Nucl Instrum Methods Phys Res B. 2017;407: 47–54. 10.1016/j.nimb.2017.05.052

[61] McDonough CA, Guelfo JL, Higgins CP. Measuring total PFASs in water: The tradeoff between selectivity and inclusivity. Curr Opin Environ Sci Health. 2019;7: 13–18. 10.1016/j.coesh.2018.08.005 33103012 PMC7584354

[62] Patlewicz G, Richard AM, Williams AJ, Grulke CM, Sams R, Lambert J, et al. A Chemical Category-Based Prioritization Approach for Selecting 75 Per- and Polyfluoroalkyl Substances (PFAS) for Tiered Toxicity and Toxicokinetic Testing. Environ Health Perspect. 2019;127: 014501. 10.1289/EHP4555 30632786 PMC6378680

[63] Sustainable Packaging for the State of California Act (Proposed Regulations). Public Resources Code. Sect. 42370 2018. Available from: https://www2.calrecycle.ca.gov/PublicNotices/Documents/11542.

[64] Michigan Science Advisory Workgroup. Health Based Drinking Water Value Recommendations for PFAS in Michigan. Report developed for the Michigan PFAS Action Response Team, Lansing, Michigan. 6 27, 2019. 2019.

[65] Patlewicz G. PFAS Prioritisation for Targeted Testing. Presented at Office of Environmental Health Hazard Assessment (OEHHA) of Cal EPA Workshop on Read-Across, Oakland, CA, May 02–03, 2019. 2019. Available from: 10.23645/epacomptox.8127137.

[66] ITRC. Naming Conventions and Physical and Chemical Properties of Per- and Polyfluoroalkyl Substances (PFAS). 2020. Available from: https://pfas-1.itrcweb.org/fact_sheets_page/PFAS_Fact_Sheet_Naming_Conventions_April2020.pdf.

[67] Arp HPH, Knutsen H. Could We Spare a Moment of the Spotlight for Persistent, Water-Soluble Polymers? Environ Sci Technol. 2019; acs.est.9b07089. 10.1021/acs.est.9b07089 31845804

[68] Naidu R, Nadebaum P, Fang C, Cousins I, Pennell K, Conder J, et al. Per- and poly-fluoroalkyl substances (PFAS): Current status and research needs. Environ Technol Innov. 2020;19: 100915. 10.1016/j.eti.2020.100915

[69] Newell CJ, Adamson DT, Kulkarni PR, Nzeribe BN, Stroo H. Comparing PFAS to other groundwater contaminants: Implications for remediation. Remed J. 2020;30: 7–26.

[70] Gouin T, Becker RA, Collot A, Davis JW, Howard B, Inawaka K, et al. Toward the Development and Application of an Environmental Risk Assessment Framework for Microplastic. Environ Toxicol Chem. 2019;38: 2087–2100. 10.1002/etc.4529 31233238 PMC6852392

[71] ECETOC. An evaluation of the challenges and limitations associated with aquatic toxicity and bioaccumulation studies for sparingly soluble and manufactured particulate substances. Technical Report no 132. 2019.

[72] Backhaus T, Wagner M. Microplastics in the Environment: Much Ado about Nothing? A Debate. Glob Chall. 2019; 1900022. 10.1002/gch2.201900022 32685194 PMC7268194

[73] Zimmermann L, Göttlich S, Oehlmann J, Wagner M, Völker C. What are the drivers of microplastic toxicity? Comparing the toxicity of plastic chemicals and particles to Daphnia magna. Environ Pollut. 2020; 115392. 10.1016/j.envpol.2020.115392 32871484

[74] Groh KJ, Backhaus T, Carney-Almroth B, Geueke B, Inostroza PA, Lennquist A, et al. Overview of known plastic packaging-associated chemicals and their hazards. Sci Total Environ. 2019;651: 3253–3268. 10.1016/j.scitotenv.2018.10.015 30463173

[75] European Chemicals Agency. Annex XV Restriction Report Proposal for a Restriction: intentionally added microplastics. Version 1.2. Helsinki, Finland; 2019 Aug. Report No.: 1.2. Available from: https://echa.europa.eu/documents/10162/05bd96e3-b969-0a7c-c6d0-441182893720.

[76] Sutton R, Sedlak M, Lin D, Sun J. Contaminants of Emerging Concern A Strategy for Future Investigations. SFEI Contribution 815. Richmond, CA: San Francisco Estuary Institute; 2017.

[77] Cousins IT, Ng CA, Wang Z, Scheringer M. Why is high persistence alone a major cause of concern? Environ Sci Process Impacts. 2019;21: 781–792. 10.1039/c8em00515j 30973570

[78] Persson LM, Breitholtz M, Cousins IT, de Wit CA, MacLeod M, McLachlan MS. Confronting Unknown Planetary Boundary Threats from Chemical Pollution. Environ Sci Technol. 2013;47: 12619–12622. 10.1021/es402501c 23980998

[79] Jahnke A, Arp HPH, Escher BI, Gewert B, Gorokhova E, Kühnel D, et al. Reducing Uncertainty and Confronting Ignorance about the Possible Impacts of Weathering Plastic in the Marine Environment. Environ Sci Technol Lett. 2017;4: 85–90. 10.1021/acs.estlett.7b00008

[80] Phillips A, Pesce A. California finds widespread water contamination of ‘forever chemicals.’ Los Angeles Times. 2019. Available from: https://www.latimes.com/politics/story/2019-10-10/california-finds-widespread-contamination-of-chemicals. Accessed 29 Feb 2020.

[81] Danish Ministry of Environment and Food. “Fødevareministeren er klar til at forbyde fluorstoffer.” (in Danish). 2019. Available from: https://mfvm.dk/nyheder/nyhed/nyhed/foedevareministeren-er-klar-til-at-forbyde-fluorstoffer/.

[82] Cousins IT, Goldenman G, Herzke D, Lohmann R, Miller M, Ng CA, et al. The concept of essential use for determining when uses of PFASs can be phased out. Environ Sci Process Impacts. 2019;21: 1803–1815. 10.1039/c9em00163h 31204421 PMC6992415

[83] Villarrubia-Gómez P, Cornell SE, Fabres J. Marine plastic pollution as a planetary boundary threat–The drifting piece in the sustainability puzzle. Mar Pol. 2018;96: 213–220. 10.1016/j.marpol.2017.11.035

[84] Andrady AL. Persistence of plastic litter in the oceans. Marine anthropogenic litter. Cham: Springer; 2015. pp. 57–72.

[85] Völker C, Kramm J, Wagner M. On the Creation of Risk: Framing of Microplastics Risks in Science and Media. Glob Chall. 2019; 1900010. 10.1002/gch2.201900010

[86] Tyree C, Morrison D. Invisibles: the Plastic inside Us. Orb Media. 2017.

[87] Sedlak M, Sutton R, Miller L, Lin D. Microplastic Strategy Update. Richmond, CA: San Francisco Estuary Institute; 2019 p. 34. Report No.: SFEI Contribution Number 951.

[88] Azoulay D, Villa P, Arellano Y, Gordon M, Moon D, Miller K, et al. Plastic and Health: The Hidden Cost of a Plastic Planet. 2019. Available from: www.ciel.org/plasticandhealth/.

[89] Jambeck JR, Geyer R, Wilcox C, Siegler TR, Perryman M, Andrady A, et al. Plastic waste inputs from land into the ocean. Science. 2015;347: 768–771. 10.1126/science.1260352 25678662

[90] Europe Plastic. Plastics—the facts 2015 an analysis of European plastics production, demand and waste data. 2017.

[91] Lebreton L, Andrady A. Future scenarios of global plastic waste generation and disposal. Palgrave Commun. 2019;5: 6. 10.1057/s41599-018-0212-7

[92] Wright SL, Kelly FJ. Plastic and Human Health: A Micro Issue? Environ Sci Technol. 2017;51: 6634–6647. 10.1021/acs.est.7b00423 28531345

[93] Burres E. Conducting Rapid Trash Assessments. 2009. Available from: https://www.waterboards.ca.gov/water_issues/programs/swamp/docs/cwt/guidance/4311b.pdf. 10.1016/j.marpolbul.2009.06.014 19635625

[94] The California Water Boards. Annual Trash Data Dive. 20 Dec 2019 [cited 28 Apr 2020]. Available from: https://www.waterboards.ca.gov/resources/data_databases/a_t_datadive.html#2018inaugural_tdd.

[95] Ocean Protection Council. Resolution of the California Ocean Protection Council On Reducing and Preventing Marine Debris. 2007 p. 4. Available from: http://www.opc.ca.gov/webmaster/ftp/pdf/docs/Documents_Page/Resolutions/MarineDebris_Resolution.pdf.

[96] Ocean Protection Council and National Ocean and Atmospheric Administration Marine Debris Program. California Ocean Litter Prevention Strategy: Addressing Marine Debris from Source to Sea. 2018 p. 48.

[97] Southern California Coastal Water Research Project. International study kicks off to standardize microplastics monitoring methods. 15 Nov 2019. Available from: https://www.sccwrp.org/news/international-study-kicks-off-to-standardize-microplastics-monitoring-methods/. [cited 2020 Apr 28].

[98] California Ocean Protection Council. OPC Science Advisory Team (OPC-SAT). [cited 2020 Apr 28]. Available from: http://www.opc.ca.gov/science-advisory-team/.

[99] Bokkers B, van de Ven B, Janssen P, Bil W, van Broekhuizen F, Zeilmaker M, et al. Per-and polyfluoroalkyl substances (PFASs) in food contact materials. 2019.

[100] Joerss H, Apel C, Ebinghaus R. Emerging per- and polyfluoroalkyl substances (PFASs) in surface water and sediment of the North and Baltic Seas. Sci Total Environ. 2019;686: 360–369. 10.1016/j.scitotenv.2019.05.363 31181522

[101] Rulis A. U.S. FDA: Agency Response Letter GRAS Notice No. GRN 000082. 2002. Available from: https://wayback.archive-it.org/7993/20171031023801/https://www.fda.gov/Food/IngredientsPackagingLabeling/GRAS/NoticeInventory/ucm154595.htm.

[102] Ateia M, Maroli A, Tharayil N, Karanfil T. The overlooked short- and ultrashort-chain poly- and perfluorinated substances: A review. Chemosphere. 2019;220: 866–882. 10.1016/j.chemosphere.2018.12.186 33395808

[103] Brennholt N, Heß M, Reifferscheid G. Freshwater microplastics: challenges for regulation and management. Freshwater Microplastics. Cham: Springer; 2018. pp. 239–272.

[104] State Water Resources Control Board. Resolution No. 2020–0021. Adoption of Definition of “Microplastics in Drinking Water.” Jun 16, 2020. Available from: https://www.waterboards.ca.gov/board_decisions/adopted_orders/resolutions/2020/rs2020_0021.pdf.

[105] World Health Organization. Microplastics in drinking-water. Geneva; 2019. Available from: http://edepot.wur.nl/498693.

[106] Shen M, Song B, Zeng G, Zhang Y, Huang W, Wen X, et al. Are biodegradable plastics a promising solution to solve the global plastic pollution? Environ Pollut. 2020;263: 114469. 10.1016/j.envpol.2020.114469 32272422

[107] Cousins IT, DeWitt JC, Glüge J, Goldenman G, Herzke D, Lohmann R, et al. Strategies for grouping per- and polyfluoroalkyl substances (PFAS) to protect human and environmental health. Environ Sci Process Impacts. 2020; 10.1039.D0EM00147C. 10.1039/d0em00147c 32495786 PMC7585739

[108] Kwiatkowski CF, Andrews DQ, Birnbaum LS, Bruton TA, DeWitt JC, Knappe DRU, et al. Scientific Basis for Managing PFAS as a Chemical Class. Environ Sci Technol Lett. 2020; acs.estlett.0c00255. 10.1021/acs.estlett.0c00255 PMC8297807 34307722

[109] Twiss MR. Standardized methods are required to assess and manage microplastic contamination of the Great Lakes system. J Great Lakes Res. 2016;42: 921–925.

[110] Hidalgo-Ruz V, Gutow L, Thompson RC, Thiel M. Microplastics in the Marine Environment: A Review of the Methods Used for Identification and Quantification. Environ Sci Technol. 2012;46: 3060–3075. 10.1021/es2031505 22321064

[111] Primpke S, Christiansen SH, Cowger W, De Frond H, Deshpande A, Fischer M, et al. Critical Assessment of Analytical Methods for the Harmonized and Cost Efficient Analysis of Microplastics. Appl Spectrosc. 2020; 000370282092146. 10.1177/0003702820921465 32249594

[112] Koelmans AA, Besseling E, Foekema E, Kooi M, Mintenig S, Ossendorp BC, et al. Risks of Plastic Debris: Unravelling Fact, Opinion, Perception, and Belief. Environ Sci Technol. 2017;51: 11513–11519. 10.1021/acs.est.7b02219 28971682 PMC5677762

